# Editorial: Second Line Treatment of Non-Small Cell Lung Cancer: Clinical, Pathological and Molecular Aspects of Novel Promising Drugs

**DOI:** 10.3389/fmed.2017.00063

**Published:** 2017-05-22

**Authors:** Umberto Malapelle, Pierlorenzo Pallante

**Affiliations:** ^1^Department of Public Health, University of Naples Federico II, Naples, Italy; ^2^Institute of Experimental Endocrinology and Oncology (IEOS) “G. Salvatore”, National Research Council (CNR), Naples, Italy

**Keywords:** NSCLC, liquid biopsy diagnostics, precision medicine, molecular markers, immunotherapy, antiangiogenic therapy

**Editorial on the Research Topic**

**Second Line Treatment of Non-Small Cell Lung Cancer: Clinical, Pathological and Molecular Aspects of Novel Promising Drugs**

The advent of precision medicine and predictive molecular pathology led to a revolution in clinical management of patients with non-small cell lung cancer. The discovery of oncogene addiction allowed the development of targeted therapies that represent newer therapeutic options reserved to those patients harboring specific gene alterations, such as EGFR mutations, ALK, and ROS1 translocations (Lazzari et al.; Sullivan and Planchard; Tran and Klempner; Köhler). In addition, the introduction of immunotherapy, with anti PD-1 Pembrolizumab, in first-line treatment of NSCLC represents the best choice for EGFR, ALK, and ROS1 wild-type patients expressing PD-L1 on ≥50% of neoplastic cells (Cortinovis et al.). Despite the survival improvement achieved with these new therapeutic options in first-line treatment, about 30% of patients do not obtain a tumor response (Lazzari et al.). Moreover, those patients, initially sensitive to these treatments, acquire resistance and develop tumor progression. Approximately 60% of the patients progressing from first-line therapy receiving further systemic treatment in the second-line setting (Lazzari et al.; Cortinovis et al.) Also in second line, the armamentarium for the treatment of patients with NSCLC, includes a pletora of new drugs, such as immune checkpoint inhibitors (Pembrolizumab, Nivolumab) (Cortinovis et al.), third generation tyrosine kinase inhibitors (Osimertinib) (Molina-Vila et al.; Zugazagoitia et al.), and anti-angiogenic agents (Nintedanib and Ramucirumab) (Corrales et al.; Manzo et al.).

This exciting therapeutic scenario for NSCLC patients still has unsolved questions and challenging issues, in particular regarding the optimal selection of the patient population through the individualization of the correct methodology and biological source of material (tissues vs liquid biopsy) for clinical relevant biomarkers assessment (Molina-Vila et al.). Probably the right way is to give all the available opportunities to patients, but challenges and pitfalls should be carefully debated.

Taken together, the papers published in Research Topic “Second Line Treatment of Non-Small Cell Lung Cancer: Clinical, Pathological and Molecular Aspects of Novel Promising Drugs” represent a critical discussion focused on the older therapies and the historical development of second line, putting into perspective the new agents available in clinical practice, defining their importance from a clinical point of view, but also to consider and exploit the complex molecular mechanisms responsible of their efficacy or of the subsequently observed resistance phenomena, to support the oncologist to design the best therapeutic strategies for NSCLC patients.

## Author Contributions

UM and PP contributed equally to this paper.

## Conflict of Interest Statement

The authors declare that the research was conducted in the absence of any commercial or financial relationships that could be construed as a potential conflict of interest.

